# Effects of Exercise Training under Hyperbaric Oxygen on Oxidative Stress Markers and Endurance Performance in Young Soccer Players: A Pilot Study

**DOI:** 10.1155/2016/5647407

**Published:** 2016-12-19

**Authors:** Carlos Burgos, Carlos Henríquez-Olguín, David Cristóbal Andrade, Rodrigo Ramírez-Campillo, Oscar F. Araneda, Allan White, Hugo Cerda-Kohler

**Affiliations:** ^1^Unidad de Fisiología Integrativa del Ejercicio, Laboratorio de Ciencias del Ejercicio, Clínica MEDS, Avenida Isabel La Católica 3740, 7550557 Santiago, Chile; ^2^Departamento de Ciencias de la Actividad Física, Núcleo de Investigación en Salud, Actividad Física y Deporte, Universidad de Los Lagos, Avenida Fuchslocher 1035, 5290000 Osorno, Chile; ^3^Laboratorio Integrativo de Biomecánica y Fisiología del Esfuerzo (LIBFE), Escuela de Kinesiología, Facultad de Medicina, Universidad de los Andes, Avenida Monseñor Álvaro del Portillo 12455, 7620001 Santiago, Chile; ^4^Laboratorio Ciencias de la Actividad Física, Instituto de Ciencias Biomédicas, Facultad de Medicina, Universidad de Chile, Avenida Independencia 1027, 8380453 Santiago, Chile; ^5^Escuela de Ciencias de la Actividad Física, el Deporte y la Salud, Facultad de Medicina, Universidad de Santiago, Avenida Libertador Bernardo O'Higgins 3363, 9170022 Santiago, Chile

## Abstract

The aim of the present study was to determine the effects of three weeks of hyperbaric oxygen (HBO_2_) training on oxidative stress markers and endurance performance in young soccer players. Participants (18.6 ± 1.6 years) were randomized into hyperbaric-hyperoxic (HH) training (*n* = 6) and normobaric normoxic (NN) training (*n* = 6) groups. Immediately before and after the 5th, 10th, and 15th training sessions, plasma oxidative stress markers (lipid hydroperoxides and uric acid), plasma antioxidant capacity (6-hydroxy-2,5,7,8-tetramethylchroman-2-carboxylic acid [TROLOX]), arterial blood gases, acid-base balance, bases excess (BE), and blood lactate analyses were performed. Before and after intervention, maximal oxygen uptake (VO_2_max) and peak power output (PPO) were determined. Neither HH nor NN experienced significant changes on oxidative stress markers or antioxidant capacity during intervention. VO_2_max and PPO were improved (moderate effect size) after HH training. The results suggest that HBO_2_ endurance training does not increase oxidative stress markers and improves endurance performance in young soccer players. Our findings warrant future investigation to corroborate that HBO_2_ endurance training could be a potential training approach for highly competitive young soccer players.

## 1. Introduction

Hyperbaric oxygen (HBO_2_) therapy is the medical administration of 100% oxygen (O_2_) at pressures higher than 1 absolute atmosphere (ATA) and during the last decade elite and competitive athletes have used HBO_2_ to accelerate the recovery after injuries or exercise-induced muscle damage [1–3].

O_2_ plays an essential role in cell metabolism and its availability is a principal determinant of maximal O_2_ uptake (VO_2_max) [4]. Acute exposure to normobaric hyperoxia (i.e., 60% to 100% O_2_) increases O_2_ availability and may improve physical performance in a dose-dependent manner [5, 6]. However, It has been shown that preexercise exposure to normobaric hyperoxia did not modify the performance during high intensity exercise [7, 8], endurance exercise [9], or resistance training [7]. Similarly, preexposure to HBO_2_ has no acute effect on physical performance [10, 11]. Furthermore, it has been suggested that long-term training in normobaric hyperoxia has no additional effect on endurance performance adaptations [12, 13]. This lack of effects could be related to O_2_ diffusion limitation during normobaric hyperoxia exposure [14]. Therefore, other hyperoxic environments, such as HBO_2_, may be more suitable for long-term endurance adaptations since this condition allows a higher O_2_ diffusion [15].

Exposures to supraphysiological O_2_ levels may increase the risk of developing systemic and cellular oxidative stress [16–18]. Besides, it has been reported that HBO_2_ induces symptoms of central nervous system toxicity as headache or nausea in ~2–6% of divers who dive between ~1.3 and 1.6 ATA with an oxygen concentration of ~91%. However, the probability of these symptoms to appear only increases after 4 h of diving [19, 20]. Thus, it is still a matter of debate if HBO_2_ training may induce harmful effects as oxidative stress [7, 21].

To our knowledge, no studies have been carried out to establish the chronic effects of intermittent HBO_2_ training on oxidative stress and endurance performance in soccer players. The aim of this pilot study was to determine the chronic effects of endurance training under HBO_2_ on oxidative stress and endurance performance. It was hypothesized that HBO_2_ training does not increase oxidative stress biomarkers and induces greater endurance adaptations compared to normobaric hyperoxia training in young soccer players.

## 2. Material and Methods

### 2.1. Experimental Approach

**Figure 1:**
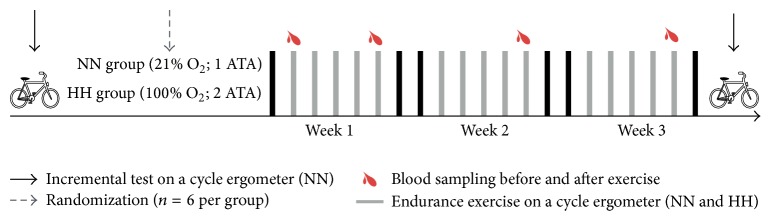
Schematic representation of training intervention. NN = normobaric normoxia; HH = hyperbaric hyperoxia. ATA = absolute atmosphere.

To compare the acute and chronic effects of HBO_2_ versus normobaric normoxia training on oxidative stress and endurance performance, the participants were randomized into a hyperbaric hyperoxia training group (HH, *n* = 6) and normobaric normoxia training group (NN, *n* = 6) during three weeks. Immediately before and after the 1st (baseline), 5th, 10th, and 15th training sessions, oxidative stress markers (plasma lipid hydroperoxides and plasma uric acid), plasma antioxidant capacity (6-hydroxy-2,5,7,8-tetramethylchroman-2-carboxylic acid [TROLOX]), arterial blood gases, acid-base balance, and lactate analysis were performed. Furthermore, before and after three weeks of training, VO_2_max and peak power output (PPO) were measured with a cycle ergometer test (Figure 1).

### 2.2. Participants

Twelve young male soccer players (18.6 ± 1.6 years) participating in the young Chilean National Soccer Championship were included in the pilot study. The participants were recruited from the same team; therefore, they had similar training/competition load and nutritional support. All measurements were carried out between 8:00 a.m. and 12:00 p.m. All participants were medically assessed and carefully informed about the experimental procedures and possible risks and benefits associated with participation in the study and signed an informed consent. The study was conducted in accordance to the Declaration of Helsinki and was approved by the ethics committee of the Faculty of Medicine, Universidad de Chile. Subject characteristics are presented in Table 1.

### 2.3. Training Program

All study was performed in a hyperbaric chamber at ~540 m above sea. Additionally to their regular training, soccer players from the HH and NN groups were submitted to 30 min of endurance exercise on a cycle ergometer (Ergomedic 828E Monark, Sweden), at 75% of power output at VO_2_max during three weeks. The NN group completed 15 endurance training sessions (5 times per week) in a hyperbaric chamber (Model C.H.10 N°4, Osorio Hnos. y Cia. Ltda., Chile) at 1 atmosphere absolute (ATA) (unpressurized chamber), breathing normoxic gas (air) (21% O_2_). HH group completed the same volume of endurance training with an additional ATA (i.e., at 2.0 ATA) in the hyperbaric chamber breathing hyperoxic gas (100% O_2_) (see schematic representation in Figure 1).

### 2.4. Cycle Ergometer Test

To determine the chronic effect of HBO_2_ on endurance performance (VO_2_max and PPO), each participant completed an incremental maximum cycle ergometer (Ergomedic 828E Monark, Sweden) test previously described [22] under normobaric normoxic conditions, 72 hours before and after the three-week training period. Briefly, the test started at a power output of 75 W and the workload was increased 25 W·min^−1^ to exhaustion. The test was finished when minimum pedal cadence could not be maintained at 70 rpm. Gas exchange was recorded continuously with a portable breath-to-breath gas analyzer (Cortex Metamax 3B, Leipzig, Germany). The analyzer was calibrated according to the manufacturer instructions prior to each trial. Pulmonary ventilation (VE), O_2_ uptake (VO_2_), expired carbon dioxide (VCO_2_), and respiratory exchange ratio (RER) were averaged over 10 s, with the highest 30 s value (i.e., three consecutive 10 s periods) used in the analysis. VO_2_max was determined according to achievement of previously established criteria [23]: (1) plateau in oxygen consumption (increase < 150 mL·min^−1^), (2) respiratory exchange ratio > 1.1, and (3) ≥90% of theoretical maximal heart rate. The VO_2_max was expressed relative to body mass (mL·kg^−1^·min^−1^) and PPO in watts (w).

### 2.5. Blood Sampling

Immediately before and after the 1st (baseline), 5th, 10th, and 15th training sessions, oxidative stress markers (plasma lipid hydroperoxides and plasma uric acid), plasma antioxidant capacity (6-hydroxy-2,5,7,8-tetramethylchroman-2-carboxylic acid [TROLOX]), arterial blood gases, and acid-base balance analyses were performed.

All plasmatic variables were analyzed from blood samples (10 mL) obtained from the brachial vein. Samples were centrifuged at 2.422 ×g (RCF) for 10 minutes (Model PLC-03, Gemmy Industrial Corp., Germany). The plasma obtained was divided into aliquots of 4 mL and transported to the laboratory in a container with crushed ice, where they were frozen at −80°C until the time of analysis.

### 2.6. Plasma Lipid Hydroperoxides

For the analysis of plasma lipid hydroperoxides a routine procedure was used [24]. Briefly, 100 *μ*L of methanol with triphenylphosphine (15 mM - inhibitor of endoperoxides) was added to 100 *μ*L of deproteinized plasma with TCA 15% and incubated for 30 min. Then 900 *μ*L of FOX version 2 was added and incubated during 45 min at room temperature. The peroxides on the sample oxidized Fe^+2^ to Fe^+3^, in an acidic medium of H_2_SO_4_ (25 mM). This process was monitored with xylenol orange (i.e., it changes from yellow to blue when it reacts with Fe^+3^) and the absorbance was evaluated with a spectrophotometer (S-20 Vis, Boeco Germany) set at 560 nm. A standard curve was constructed with H_2_O_2_.

### 2.7. Plasma Uric Acid

The used method was based upon the oxidation of uric acid to alatoine and H_2_O_2_ by the uricase enzyme. H_2_O_2_ reacts with 3-5-dichloro-2-hydroxy-bencesulfonic and 4-aminoantipyrine by the peroxidase (Valtek Diagnostics, Santiago, Chile). Briefly, 1.0 mL of uricase and peroxidase was added to 25 *μ*L of plasma and uric acid (10 mg/dL). Then, the solution was stirred vigorously and incubated for 5 min at 37°C. The absorbance of the sample at 520 nm was evaluated using a spectrophotometer (S-20 Vis, Boeco Germany).

### 2.8. Plasma Antioxidant Capacity

Plasma antioxidant capacity was determined with a 1250 Luminometer (LKB-Wallac, Turku, Finland) and according to previously described methods [25, 26]. Briefly, using the chemiluminescence technique to measure the antioxidant capacity of biological fluids, the light emission occurs when the chemiluminescent substrate (i.e., luminol) is oxidized by H_2_O_2_ in a reaction catalyzed by “horseradish peroxidase.” The stabilities and intensities of the light emitted are high due to the addition of p-iodine phenol. Its emission depends on the constant production of intermediate free radicals derivative of p-iodine phenol, luminal, and O_2_. For this reason, the light emission is sensitive for antioxidants but is reestablished when these antioxidants are consumed in the reaction. As the generation of intermediate free radicals is constant, the light suppression period is directly related to the amount of antioxidant in the sample. The assessment is sensitive enough to measure the antioxidant capacity of biological samples, being expressed relatively to the TROLOX, soluble analogous of the vitamin E. The plasma antioxidant capacity results are expressed as an equivalent of TROLOX per liter of sample (*μ*mol/L).

### 2.9. Blood Lactate

For lactate measurements, capillary blood was obtained from earlobe and assessed with reagent strips (BM-Lactate, Roche Diagnostics, Germany) [27] and a portable handheld lactate meter (Accusport®, Boehringer Mannheim, Germany).

### 2.10. Blood Gases and Acid-Base Analysis

Plasmatic variables were analyzed from blood samples obtained from the brachial vein. O_2_ partial pressure (PO_2_), bicarbonate (HCO_3_^−^), base excess (BE), carbon dioxide partial pressure (PCO_2_), and pH were determined using a portable gas analyzer (i-STAT, Abbot Lab. USA). All the results were normalized to body temperature. HCO_3_^−^ and BE were determined in relation to pH and PCO_2_ by the Henderson-Hasselbalch equation. The PO_2_ was determined through oxidation current, which is proportional to O_2_ dissolved in blood.

### 2.11. Statistical Analysis

Descriptive statistics (mean ± SD) for the different variables were calculated. Shapiro-Wilk normality test for the dependent variables data was used to determine if the distribution was normal. According to results shown by Impellizzeri et al. [28], the sample size (*n* = 6 per group) allows identification of a difference of 5.4 mL·kg^−1^·min^−1^ in VO_2_max between groups, assuming a statistical power of 80%.

The practical significance of data was assessed via magnitude-based inferences [29]. Because the formula for Cohen's *d* gives a biased estimate of the population effect size, especially for small samples (*n* < 20), effect sizes (ES, 90% CI) were calculated through the* corrected effect size*, or Hedges's *g* [30]. The following threshold values for ES were employed: <0.2 as trivial, >0.2 as small, >0.6 as moderate, >1.2 as large, >2.0 as very large, and 4.0 as extremely large [31]. Traditional statistical approach was assessed using Kruskal-Wallis test followed by Dunn's post hoc analysis and 2-way ANOVA followed by Tukey post hoc analysis. The *α* level was set at *p* < 0.05 for statistical significance. All statistical analysis was performed using GraphPad Prism 6.0 (GraphPad Software, Inc., San Diego, CA, USA).

## 3. Results

Before intervention no differences were found between groups for descriptive characteristics (Table 1).

In order to clarify if chronic or acute HBO_2_ exercise training increases oxidative stress, plasma lipid hydroperoxides, plasma uric acid, and plasma antioxidant capacity (TROLOX) were measured once a week during the training program (see Material and Methods). No differences in oxidative stress markers were observed in HH and NN during intervention (*p* > 0.05, Table 2). ES showed unclear effect in all variables either in basal or postexercise values during intervention.

To analyze changes in acid-base balance, a blood sample was obtained immediately before and after the 1st and 15th endurance training sessions (Table 3). Plasmatic HCO_3_^−^ (−27%, *p* = 0.001; −30%, *p* = 0.0003, 1st and 15th session, resp.) and BE (−145%, *p* = 0.001; −179%, *p* = 0.0004, 1st and 15th session, resp.) were significantly reduced after training sessions only in the NN group (Table 3). ES shows unclear effect in all variables either in pre-post basal or pre-post exercise values during intervention.

A low concentration of blood lactate in normobaric hyperoxia or HBO_2_ conditions has been reported in many studies [19, 20, 24, 26]. Plasma lactate was significantly increased after training sessions (181%, *p* = 0.0002; 134%, *p* = 0.001, 1st and 15th session, resp.) only in NN group (Table 3). Furthermore, postexercise plasma lactate was significantly higher in NN versus HH group after training sessions (135%, *p* = 0.004; 162%, *p* = 0.029, 1st and 15th session, resp.) (Table 3). ES shows unclear effect in all variables either in pre-post basal or pre-post exercise values during intervention.

To determine the endurance adaptations to HBO_2_ training, participants performed an incremental test before and after experimental intervention. Traditional statistical approach shows no significant differences between groups in VO_2_max or PPO before and after training (Figure 1). However, ES shows an unclear effect and a moderate effect in VO_2_max and PPO for NN and HH groups, respectively.

## 4. Discussion

The main findings of the present study suggest that 3 weeks of exercise training in HBO_2_ condition do not increase oxidative stress markers and seem to improve endurance capacity compared to normobaric normoxia training in young soccer players.

Several diseases have been associated with oxidative stress and others with free radical production. In skeletal muscle, exercise induces an increase of mitochondrial and nonmitochondrial [32] reactive oxygen species (ROS) generation. Some studies have shown that high dietary antioxidant intake is associated with an impair in the exercise-induced adaptive gene expression [33, 34]. Thus, during the last decade ROS molecules have emerged as key players in the molecular response to exercise.

Some animal and human data have suggested that breathing supraphysiological O_2_ exposure may increase ROS production and induce oxidative stress [16, 17, 35]. However, the HBO_2_-induced DNA damage rapidly disappears after the end of the HBO_2_ exposure in healthy humans [36]. Moreover, DNA damage was detected only after the first treatment and not after further treatments under the same conditions, indicating an increase in antioxidant defense [36]. In the present study, we showed that acute and chronic training in HBO_2_ environment did not modify lipid peroxidation or plasma antioxidant capacity. This is in accordance with previous studies where no changes in lipid peroxidation or oxidative stress marker were reported in patients with various pathologies [37]. Moreover, Alcaraz-García et al. reported that, in professional divers, HBO_2_ training (~1.7 ATA; 100% O_2_) for 2-3 times per week during 12 weeks did not induce an important oxidative hyperoxia-induced stress due an adaptive process [38]. Taken together, these data suggest that training in a HBO_2_ environment does not modify oxidative stress in human volunteers.

Many studies have reported an increase of antioxidant defense after HBO_2_ exposure [36, 37, 39]. In our study the antioxidant plasma capacity was unaffected during three weeks of HBO_2_ training. Possibly, intracellular changes in antioxidant defense may mediate some of our results [40]. Future studies should determine the mechanisms involved in this response to HBO_2_.

**Figure 2:**
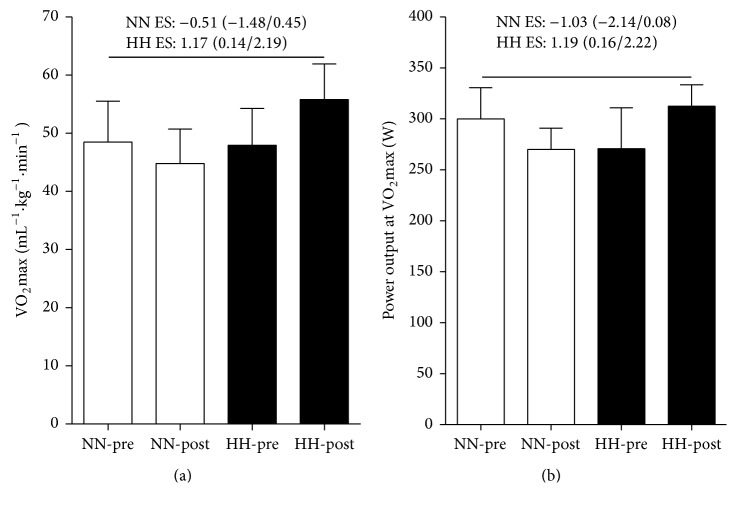
Effects of 3 weeks of NN and HH endurance training on VO_2_max (a) and peak power output (b) in young soccer players. ES: effect size. NN = normobaric normoxia; HH = hyperbaric hyperoxia.

VO_2_max is a relevant physiological variable in soccer, where significant relationships between VO_2_max and the total distance covered during games, time spent at high intensity, and team classification during tournaments had been reported [41]. It has been shown that, compared to normobaric normoxia, acute breathing of normobaric hyperoxia gas during exercise enhances performance [13, 42–44]; however, chronic exercise under normobaric hyperoxia did not improve VO_2_max more than under normobaric normoxia training [12, 13, 45]. Furthermore, preexposure to HBO_2_ did not increase the subsequent exercise performance [11]. In the present study we show that three weeks of endurance training under HBO_2_ seems to induce an increase in VO_2_max and PPO (Figure 2). It is possible that training under HBO_2_ conditions increases the alveolar-capillary O_2_ exchange [15, 46]. However, more studies are needed to clarify this point. Considering that training environment can affect physiological adaptations (i.e., specificity effect), it is interesting to note that although VO_2_max was measured under normobaric normoxia (i.e., an environment specific to participants from the NN endurance training group) only soccer players who had practiced under HBO_2_ achieved a significant increase in this variable, highlighting the positive effect of HBO_2_ training on endurance performance.

The exact mechanisms involved in the increase of PPO during hyperoxia are not completely understood. However, the increased VO_2_max, lower lactate, and acid-base balance under HBO_2_ conditions may play a significant role. In this way, acute changes in plasma HCO_3_^−^ were smaller in acute response to exercise in HH versus NN, suggesting that metabolic buffering is less involved in HBO_2_ training. This could be related to lactate metabolism, because it has been reported that transport of lactate across the plasma membrane of skeletal muscle fibers involves a proton-linked monocarboxylate transporter, playing an important role in the pH regulation of skeletal muscle and extracellular fluids [47].

## 5. Conclusions

HBO_2_ training does not increase systemic oxidative stress markers and seems to improve endurance capacity under normobaric normoxia environment in young soccer players. Therefore, our findings warrant future investigation to corroborate that HBO_2_ endurance training, or other models of training as high intensity interval training and resistance training, may be an interesting training approach for highly competitive young soccer players to increase their competitive performance. Moreover, training in HBO_2_ conditions can be an alternative for injured athletes who need a quick return to competition.

## Figures and Tables

**Table 1:** Baseline characteristics of the study participants.

	NN group (*n* = 6)	HH group (*n* = 6)
Age (years)	18.5 ± 1.6	18.8 ± 1.6
Height (cm)	175.8 ± 2.7	177.5 ± 2.3
Body mass (kg)	68.1 ± 6.4	71.7 ± 2.3
Body fat mass (%)	14.4 ± 4.0	13.9 ± 1.9
Body muscle mass (%)	43.9 ± 2.9	45.0 ± 2.5

Data is shown as mean ± SD. NN = normobaric normoxia. HH = hyperbaric hyperoxia.

**Table 2:** Plasma oxidative stress markers and antioxidant capacity immediately before (pre) and after (post) the 5th, 10th, and 15th training session during 3 weeks of hyperbaric hyperoxia (HH) and normobaric normoxia (NN) endurance training.

		Baseline	5th session	10th session	15th session	Pre-post ES (90% CI)	Rating
NN							
Plasma uric acid (mg/dL)	Pre	4.5 ± 0.2	6.8 ± 3.4	5.7 ± 3.2	5.9 ± 3.4	0.5 (−0.4/1.5)	Unclear
	Post	5.0 ± 0.7	6.4 ± 3.7	5.1 ± 2.3	5.8 ± 1.8	0.5 (−0.5/1.5)	Unclear
Plasma lipid hydroperoxides (*μ*mol/L)	Pre	6.2 ± 2.3	7.3 ± 3.5	8.7 ± 5.1	6.5 ± 4.7	0.1 (−0.9/1.0)	Unclear
	Post	9.1 ± 4.7	8.2 ± 4.5	10.3 ± 3.9	8.5 ± 4.7	−0.1 (−1.1/0.8)	Unclear
TROLOX (*μ*mol/L)	Pre	207 ± 76.9	222 ± 112	188 ± 105	192 ± 113	−0.1 (−1.1/0.8)	Unclear
	Post	183 ± 83.1	210 ± 123	165 ± 75.8	190 ± 61.3	0.1 (−0.9/1.0)	Unclear

HH							
Plasma uric acid (mg/dL)	Pre	6.0 ± 1.6	6.3 ± 1.6	6.3 ± 1.7	6.1 ± 0.2	0.1 (−0.8/1.1)	Unclear
	Post	5.7 ± 2.0	7.3 ± 1.9	6.7 ± 1.5	6.0 ± 1.1	0.2 (−0.8/1.1)	Unclear
Plasma lipid hydroperoxides (*μ*mol/L)	Pre	5.6 ± 3.9	5.6 ± 3.9	6.4 ± 3.7	5.9 ± 3.4	0.1 (−0.9/1.0)	Unclear
	Post	5.4 ± 3.3	5.4 ± 3.3	6.5 ± 3.1	8.4 ± 3.2	0.9 (−0.1/1.9)	Unclear
TROLOX (*μ*mol/L)	Pre	207 ± 34.7	205 ± 54.1	207 ± 57.9	200 ± 9.5	−0.3 (−1.2/0.7)	Unclear
	Post	219 ± 18.4	238 ± 61.7	219 ± 48.7	195 ± 36.9	−0.8 (−1.7/0.2)	Unclear

Data is shown as mean ± SD. TROLOX: plasma antioxidant capacity.

**Table 3:** Acid-base balance immediately before (pre) and after (post) training session of 3 weeks of hyperbaric hyperoxia (HH) and normobaric normoxia (NN) endurance training.

		Baseline	15th session	Pre-post ES (90% CI)	Rating
NN					
PCO_2_ (mmHg)	Pre	56.5 ± 8.0	55.5 ± 7.2	−0.1 (−1.1/0.8)	Unclear
	Post	39.2 ± 7.8^*∗*^	37.3 ± 2.1^*∗*^	−0.3 (−1.3/0.6)	Unclear
PO_2_ (mmHg)	Pre	24.2 ± 7.4	22.0 ± 8.2	−0.3 (−1.2/0.7)	Unclear
	Post	58.7 ± 19.5^*∗*^	63.0 ± 11.4^*∗*^	0.2 (−0.7/1.2)	Unclear
pH	Pre	7.32 ± 0.04	7.34 ± 0.03	0.5 (−0.4/1.5)	Unclear
	Post	7.34 ± 0.02	7.33 ± 0.10	−0.1 (−1.1/0.8)	Unclear
Base excess (mmol/L)	Pre	3.3 ± 1.8	3.1 ± 1.5	−0.1 (−1.1/0.8)	Unclear
	Post	−4.8 ± 3.1^*∗α*^	−5.6 ± 5.1^*∗α*^	−0.2 (−1.1/0.8)	Unclear
Bicarbonate (mmol/L)	Pre	29.3 ± 1.9	29.1 ± 1.6	−0.1 (−1.1/0.8)	Unclear
	Post	21.3 ± 3.3^*∗α*^	20.3 ± 3.9^*∗*^	−0.3 (−1.2/0.7)	Unclear
Lactate (mmol/L)	Pre	2.1 ± 0.2	2.3 ± 0.4	0.6 (−0.4/1.6)	Unclear
	Post	6.0 ± 1.5^*∗α*^	5.5 ± 1.7^*∗α*^	−0.3 (−1.2/0.7)	Unclear

HH					
PCO_2_ (mmHg)	Pre	52.8 ± 5.9	53.1 ± 5.7	0.0 (−0.9/1.0)	Unclear
	Post	48.7 ± 8.6	49.4 ± 14.4	0.1 (−0.9/1.0)	Unclear
PO_2_ (mmHg)	Pre	32.0 ± 12.6	24.5 ± 10.6	−0.6 (−1.6/0.4)	Unclear
	Post	37.2 ± 16.6	31.0 ± 16.9	−0.3 (−1.3/0.6)	Unclear
pH	Pre	7.40 ± 0.03	7.40 ± 0.01	0.0 (−0.9/0.9)	Unclear
	Post	7.40 ± 0.04	7.36 ± 0.04	−1.0 (−2.1/0.0)	Unclear
Base excess (mmol/L)	Pre	4.0 ± 2.2	4.0 ± 2.8	0.0 (−0.9/0.9)	Unclear
	Post	2.3 ± 2.8	2.5 ± 4.9	0.0 (−0.9/1.0)	Unclear
Bicarbonate (mmol/L)	Pre	29.5 ± 2.1	29.5 ± 3.5	0.0 (−0.9/0.9)	Unclear
	Post	27.7 ± 3.2	27.5 ± 4.9	0.0 (−1.0/0.9)	Unclear
Lactate (mmol/L)	Pre	1.7 ± 0.7	1.3 ± 0.1	−0.7 (−1.7/0.2)	Unclear
	Post	2.5 ± 0.7	2.1 ± 0.1	−0.7 (−1.7/0.2)	Unclear

Data is shown as mean ± S.D. *∗* denotes statistical difference with the pre value (*p* < 0.05). *α* denotes statistical difference with the HH group (*p* < 0.05). PCO_2_ and PO_2_ = partial pressure of carbon dioxide and oxygen, respectively. Pre: resting condition. Post: immediately postexercise levels.
